# Effects of hysterectomy with simultaneous bilateral salpingectomy on the subsequent pelvic mass

**DOI:** 10.1186/s13048-019-0504-6

**Published:** 2019-03-27

**Authors:** Xiaopei Chao, Xiaoxia Wang, Yu Xiao, Mingliang Ji, Shu Wang, Honghui Shi, Qingbo Fan, Lan Zhu, Jinhua Leng, Dawei Sun, Jinghe Lang

**Affiliations:** 1 Department of Obstetrics and Gynecology, Peking Union Medical College Hospital, Peking Union Medical College & Chinese Academy of Medical Science, Shuaifuyuan No. 1, Dongcheng District, Beijing, 100730 China; 2 Department of Gynecology, Gansu Provincial Maternity and Child-care Hospital, Lanzhou City, Gansu Province People’s Republic of China; 3 Department of Pathology, Peking Union Medical College Hospital, Peking Union Medical College & Chinese Academy of Medical Science, Beijing, 100730 China

**Keywords:** Hysterectomy, Salpingectomy, Pelvic mass, Diagnosis

## Abstract

**Objectives:**

To analyze the clinicopathological characteristics of subsequent pelvic masses after hysterectomy for benign diseases, and to compare the masses following hysterectomy with or without simultaneous bilateral salpingectomy.

**Methods:**

This study retrospectively analyzed patients undergone reoperation for pelvic mass subsequently to previous hysterectomy for benign disease from January 2012 to December 2016 in Peking Union Medical College Hospital.

**Results:**

A total of 247 patients were enrolled in this study, of which 80.16% (*n* = 198) received simple hysterectomy, and 5.67% (*n* = 14) underwent hysterectomy with bilateral salpingectomy. The clinicopathological data of patients undergone simple hysterectomy or simultaneous bilateral salpingectomy was compared. In the former group, we found that 68.18% (*n* = 135) of the pelvic massed were benign, and the remaining 31.82% (*n* = 63) were malignant. In latter group, 57.10% (*n* = 8) were benign (8%) and 42.90% (*n* = 6) were malignant. Univariate analysis showed that the age of surgery for pelvic masses in patients undergoing hysterectomy with simultaneous bilateral salpingectomy was significantly younger than that in patients without salpingectomy (median, 44.5 vs 50 years, *P* < 0.0001), and the time interval between hysterectomy and onset of pelvic masses was also significantly shorter (median, 2 vs 5 years, *P* < 0.0001). And the probability of pelvic encapsulated effusion was significantly higher for the salpingectomy group. Multivariate analysis showed that there was no significant difference of the age of resection of pelvic mass, the time interval hysterectomy and pelvic mass onset, and the probability of pelvic encapsulated effusion between the two groups.

**Conclusions:**

The results showed that the incidence of secondary benign pelvic masses may be reduced when hysterectomy was performed with simultaneous bilateral salpingectomy. However, there was no statistical difference in the clinical characteristics and pathological types of pelvic masses between patients received hysterectomy with or without salpingectomy.

## Introduction

Hysterectomy is the most common gynecological operation and is widely accepted as the final treatment of many common gynecological diseases. However, the risk of reoperation increased due to complications of hysterectomy, such as pelvic adhesion. The recurrence of pelvic mass after hysterectomy is the common reason for reoperation [1–3]. It had been reported that simultaneous bilateral salpingectomy during hysterectomy may reduce the incidence of ovarian serous carcinoma and some benign pelvic diseases, thus reducing the risk of reoperation after hysterectomy [4–6]. Simultaneous bilateral salpingectomy has been routinely carried out in many medical institutions at present. However, few studies have explained whether salpingectomy affects the time of pelvic mass onset, clinical characteristics and pathological types of pelvic masses after hysterectomy in patients who have been treated again for various pelvic masses.

This study reviewed the medical records of patients received reoperation for pelvic masses following hysterectomy due to benign diseases, compared the clinical and pathological features in patients who underwent hysterectomy with or without bilateral salpingectomy, and analyzed the possible influence of simultaneous salpingectomy on the recurrence of pelvic masses.

## Methods and materials

### Ethics

This study was approved by the Ethics Committee of Peking Union Medical College Hospital (No. S-K331).

### Patients

Identify patients who underwent surgery in Peking Union Medical College Hospital for pelvic mass after hysterectomy due to benign disease from January 2012 to December 2016 using ICD-9 disease code.

#### Inclusion criteria

A history of hysterectomy due to gynecological benign disease was clearly identified, and patients who underwent reoperation for pelvic mass during the required time period were included.

#### Exclusion criteria

Patients who underwent surgery for pelvic mass but had no history of hysterectomy, had a uterine pathology of malignant tumors, or whose surgery of pelvic mass was not performed in the specified period were excluded from the study.

Medical staff collected the medical records of enrolled patients in detail, including age of hysterectomy and the indications, age of the pelvic mass onset, time interval from the hysterectomy to the onset of pelvic mass, oviduct or ovary being resected or remained in previous surgery, main manifestations and imaging features at the time of pelvic mass onset, and pathological type of the pelvic mass. Patients were divided into two groups according to hysterectomy with or without bilateral salpingectomy, and the clinicopathological characteristics of two groups were compared and analyzed.

## Statistics

**Fig. 1:**
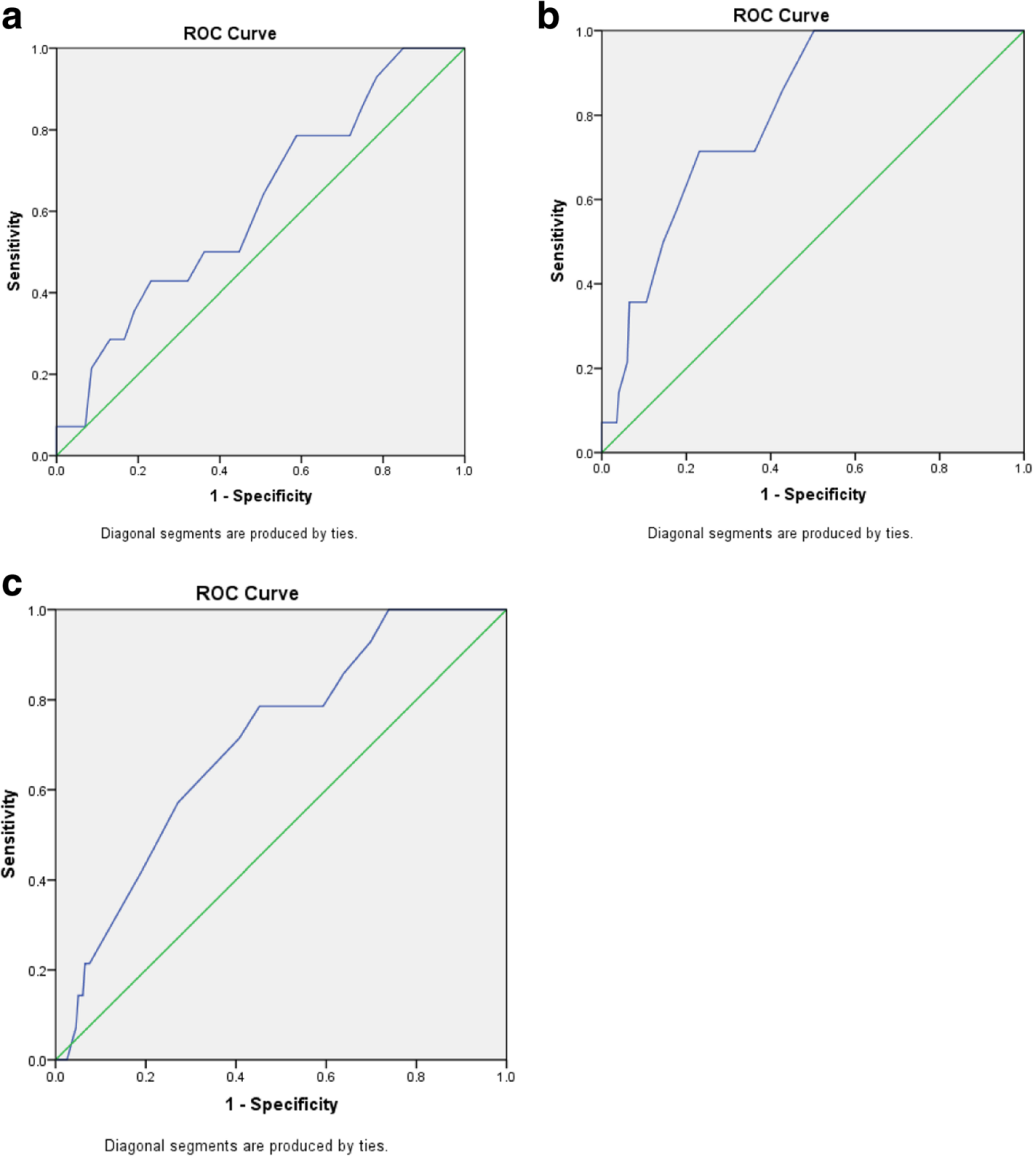
**a** ROC curve of the age of hysterectomy; **b** ROC curve of the age of pelvic masses resected; **c** ROC curve of the time interval from hysterectomy to the pelvic mass onset

The statistical analysis was performed using SPSS 23.0 (SPSS Inc., Chicago, IL, USA) in reference to whether the fallopian tubes were resected or not. Receiver Operating Characteristic (ROC) curve was constructed to define the optimal cutoff value for age of hysterectomy (< 45 years vs ≥45 years), age of the operation for pelvic mass (< 52 years vs ≥52 years), and time interval from the hysterectomy to the onset of pelvic mass (< 5 years vs ≥5 years) (Table 1, Fig. 1). Continuous variables were summarized with medians and interquartile ranges. Two groups were analyzed by independent t test, and multiple groups were analyzed by one-way ANOVA. The categorical variables were summarized with rates using Chi-square test or Fisher’s exact test to analyze. Variables with *P* < 0.05 by univariate analysis were included in multivariate analysis, and logistic regression model was used for fitting with *P* < 0.05 considered statistical significance. All analysis was two-sided and significance level was set at *P* < 0.05.

**Table 1:** Univariate analysis of clinical characteristics of pelvic masses following hysterectomy with or without simultaneous bilateral salpingectomy

	Hys N(%)	Hys + BS N(%)	*P* value
Total number of cases	198	14	
Age at hysterectomy (median[IQR], years)	43 [40,47]	42 [36,44]	0.143
Group of age at hysterectomy			
< 45	116 (58.6)	11 (78.6)	0.168
≥45	82 (41.4)	3 (21.4)	
Etiology of hysterectomy			
Endometriosis/adenomyosis			
No	150 (75.8)	4 (28.6)	< 0.0001
Yes	48 (24.2)	10 (71.4)	
Uterine leiomyoma			
No	64 (32.3)	9 (64.3)	0.020
Yes	134 (67.7)	5 (35.7)	
Postpartum massive hemorrhage			
No	195 (98.5)	14 (100)	1.0
Yes	3 (1.5)	0 (0)	
Cervical intraepithelial neoplasia			
No	186 (93.9)	14 (100)	1.0
Yes	12 (6.1)	0 (0)	
Endometrial intraepithelial neoplasia			
No	194 (98.0)	14 (100)	1.0
Yes	4 (2.0)	0 (0)	
Manifestation for medical consultation			
Physical examination findings			
No	96 (48.5)	8 (57.1)	0.531
Yes	102 (51.5)	6 (42.9)	
Abdominal distension and anorexia			
No	163 (82.3)	13 (92.9)	0.473
Yes	35 (17.7)	1 (7.1)	
Abnormal defecation			
No	190 (96.0)	13 (92.9)	0.466
Yes	8 (4.0)	1 (7.1)	
Abdominal pain			
No	151 (76.3)	9 (64.3)	0.339
Yes	47 (23.7)	5 (35.7)	
Micturition			
No	187 (94.4)	14 (100)	1.0
Yes	11 (5.6)	0 (0)	
Lower limb pain			
No	196 (99.0)	14 (100)	1.0
Yes	2 (1.0)	0 (0)	
Vaginal flow or bleeding			
No	194 (98.0)	14 (100)	1.0
Yes	4 (2.0)	0 (0)	
Total/Subtotal hysterectomy			
Total	166 (83.8)	14 (100)	0.135
Subtotal	32 (16.2)	0 (0)	
Age of surgery for pelvic mass (median[IQR])	50 [47,56]	44.5[41, 49]	< 0.0001
Age group			
< 52	114 (57.58)	14 (100)	0.001
≥52	84 (42.42)	0 (0)	
The time interval from hysterectomy to the pelvic mass onset (median[IQR])	5 [2,10]	2 [1,4]	< 0.0001
Time group			
< 5	89 (44.95)	11 (78.6)	0.024
≥ 5	109 (55.05)	3 (21.4)	
The time interval from hysterectomy to the pelvic benign mass onset (median[IQR])	4.0[2, 9.25] (*n* = 134)	1.5[0.7,3.0] (n = 8)	< 0.0001
The time interval from hysterectomy to the pelvic malignant mass onset (median[IQR])	7 [3,10.75] (*n* = 64)	4.5 [0.93, 8.25] (*n* = 6)	0.223

*Abbreviations*: *BS* bilateral salpingectomy, *Hys* Hysterectomy, *IQR* interquartile range

## Results

### Demographics data of study population

Our study included 247 patients, of whom 80.16% (*n* = 198) received simple hysterectomy, 5.67% (*n* = 14) underwent hysterectomy with bilateral salpingectomy, 12.96% (*n* = 32) hysterectomy with unilateral salpingo-oophorectomy, 1.21% (n = 3) hysterectomy with bilateral salpingo-oophorectomy. In the group of simple hysterectomy, 166 cases (83.8%) performed total hysterectomy and 32 cases (16.2%) performed subtotal hysterectomy; while in the group of hysterectomy with simultaneous bilateral salpingectomy, all of the 14 cases were performed the procedure of total hysterectomy. The clinicopathological data of patients undergone simple hysterectomy or simultaneous bilateral salpingectomy was compared. Patients who undergone simple hysterectomy, 68.18% (*n* = 135) of the pelvic massed were benign, and the remaining 31.82% (*n* = 63) were malignant. In benign masses, ovarian origin accounted for 70.37%, oviduct origin 20.00%, encapsulated effusion 10.37%; in malignant masses, ovarian origin 76.19%, pelvic extraovarian malignancy 12.70%, peritoneal cancer 4.76%, gastrointestinal derived 4.76%. Patients who undergone hysterectomy with bilateral salpingectomy, 57.14% (*n* = 8) were benign and 42.86% (n = 6) were malignant. In benign masses, ovarian origin accounted for 37.50%, encapsulated effusion 50%, extraovarian benign mass 12.5%; in malignant group, ovarian origin 83.3%, and pelvic exogenous derived 16.7%.

### Clinical characteristics between two groups

Univariate analysis showed that the age of surgery for pelvic mass in patients undergoing hysterectomy with simultaneous salpingectomy was significantly younger than those without salpingectomy [median(IQR), 44.5 (41.0, 49.0) years vs 50.0 (47.0, 56.0), *P* < 0.0001]. Grouped by ROC curve, more patients below 52 years old with pelvic mass were found in salpingectomy group (100% vs 57.58%, *P* = 0.001). The time interval from hysterectomy to the pelvic mass onset was significantly shorter (median, 2 vs 5 years, *P* < 0.0001) and more patients had the time interval less than 5 years in salpingectomy group. Further statistics were performed separately referring to the time interval from hysterectomy to the onset of benign and malignant pelvic masses. Results revealed that the time interval between benign pelvic masses and hysterectomy was shorter in salpingectomy group [median(IQR), 1.5 (0.7,3.0) vs 4.0 (2, 9.25), *P* < 0.0001]. On the contrary, there was no difference between the two groups in the time interval between hysterectomy and malignant pelvic masses onset. In addition, the cause of hysterectomy being endometriosis or adenomyosis was significantly more common in the salpingectomy group (71.4% vs 24.2%, *P* < 0.0001), while hysterectomy due to leiomyoma was less common (35.7% vs 67.7%, *P* = 0.020). There were no significant differences in age of hysterectomy and the main complaints of pelvic mass (Table 1). Multivariate analysis showed that there was no significant difference in the time interval from pelvic mass onset to the hysterectomy between two groups. Thus, in the salpingectomy group, the etiology for hysterectomy being endometriosis or adenomyosis may be related to the earlier onset of pelvic masses (Table 2).

**Table 2:** Multivariate analysis of clinical characteristics of pelvic mass after hysterectomy with or without bilateral salpingectomy

Parameter	β	S.E.	*P* value	OR(95%CI)
Intercept	−2.404	0.644	<.0001	
Endometriosis/adenomyosis	1.511	0.640	0.018	4.532 (1.293–15.880)
Leiomyoma	−0.777	0.651	0.206	0.460 (0.138–1.535)
Age of resection of pelvic mass	−1.523	0.832	0.067	0.218 (0.043–1.114)
Time interval between hysterectomy and pelvic mass onset	−0.237	0.741	0.750	0.789 (0.185–3.373)

Notes: In multivariate analysis, the equation is of significance when the *p* value of the whole test less than 0.0001

### Pathology characteristics between two groups

The pathology characteristics were compared in patients undergone hysterectomy with and without bilateral salpingectomy. Univariate analysis showed that the incidence of pelvic encapsulated effusion was higher in the salpingectomy group (28.6% vs 7.1%, *P* = 0.021), while there was no significant difference between the two groups referring to other pathological types (Table 3). However, when the main indications of hysterectomy (endometriosis/adenomyosis and leiomyoma) were included in analysis, multivariate analysis showed that there was no significant difference of the incidence of pelvic encapsulated effusion between the two groups (Table 4). This may be explained by the previous history of endometriosis or adenomyosis in the salpingectomy group.

**Table 3:** Univariate analysis of pathological characteristics of pelvic mass after hysterectomy with or without simultaneous bilateral salpingectomy

	Hys N(%)	Hys + BS N(%)	*P* value
Total number of cases	198	14	
Malignant			
No	135 (68.2)	8 (57.1)	0.391
Yes	63 (31.8)	6 (42.9)	
Ovarian serous carcinoma			
0	175 (88.4)	11 (78.57)	0.388
1	23 (11.6)	3 (21.43)	
Ovarian mucous carcinoma			
0	190 (96.0)	14 (100)	1.0
1	8 (4.0)	0 (0)	
Ovarian endometrioid carcinoma			
0	194 (98.0)	14 (100)	1.0
1	4 (2.0)	0 (0)	
Ovarian clear cell carcinoma			
0	195 (98.5)	13 (92.9)	0.241
1	3 (1.5)	1 (7.1)	
Ovarian sarcoma			
0	196 (99.0)	13 (92.9)	0.186
1	2 (1.0)	1 (7.1)	
Ovarian undifferentiated carcinoma			
0	196 (99.0)	14 (100)	1.0
1	2 (1.0)	0 (0)	
Ovarian primary PNET			
0	198 (100)	14 (100)	1.0
1	0 (0)	0 (0)	
Other uncertain types of ovarian carcinoma			
0	192 (96.97)	14 (100)	1.0
1	6 (3.0)	0 (0)	
Pelvic extraovarian carcinoma			
0	190 (96)	13 (92.9)	0.466
1	8 (4)	1 (7.1)	
Peritoneal derived carcinoma			
0	195 (98.5)	14 (100)	1.0
1	3 (1.5)	0 (0)	
Vaginal stump carcinoma			
0	197 (99.5)	14 (100)	1.0
1	1 (0.5)	0 (0)	
Carcinoma in other sites			
0	195 (98.5)	14 (100)	1.0
1	3 (1.5)	0 (0)	
Benign			
No	63 (31.8)	6 (42.9)	0.391
Yes	135 (68.2)	8 (57.1)	
Cervical leiomyoma			
0	194 (98.0)	14 (100)	1.0
1	4 (2.0)	0 (0)	
Cervical intraepithelial neoplasia			
0	197 (99.5)	14 (100)	1.0
1	1 (0.5)	0 (0)	
Hydrosalpinx			
0	178 (89.9)	14 (100)	0.371
1	20 (10.0)	0 (0)	
Mesenchymal cyst of oviduct			
0	191 (93.9)	14 (100)	1.0
1	7 (6.1)	0 (0)	
Ovarian oviduct abscess			
0	196 (99.0)	14 (100)	1.0
1	2 (1.0)	0 (0)	
Ovarian endometriosis			
0	168 (84.8)	12 (85.7)	1.0
1	30 (15.2)	2 (14.3)	
Ovarian physiological cysts			
0	178 (89.9)	14 (100)	1.0
1	20 (10.1)	0 (0)	
Ovarian serous cystadenoma			
0	184 (92.9)	13 (92.8)	1.0
1	14 (7.1)	1 (7.2)	
Ovarian mucinous cystadenoma			
0	178 (89.9)	14 (100)	0.370
1	20 (10.1)	0 (0)	
Ovarian fibroma			
0	194 (98.0)	14 (100)	1.0
1	4 (2.0)	0 (0)	
Ovarian teratoma			
0	195 (98.5)	14 (100)	1.0
1	3 (1.5)	0 (0)	
Ovarian epidermoid cyst			
0	196 (99.0)	14 (100)	1.0
1	2 (1.0)	0 (0)	
Encapsulated effusion			
0	184 (92.9)	10 (71.4)	0.021
1	14 (7.1)	4 (28.6)	
Intravascular leiomyomatosis			
0	196 (99.0)	13 (92.9)	0.186
1	2 (1.0)	1 (7.1)	
Pelvic leiomyoma			
0	196 (99.0)	14 (100)	1.0
1	2 (1.0)	0 (0)	
Others benign masses arising from extraovarian sites			
0	192 (97.0)	14 (100)	1.0
1	6 (3.0)	0 (0)	

*Abbreviations*: *BS* bilateral salpingectomy, *Hys* Hysterectomy, *IQR* interquartile range, *PNET* primitive neuroectodermal tumor

**Table 4:** Multivariate analysis of pathological characteristics of pelvic mass after hysterectomy with or without bilateral salpingectomy

Parameter	β	S.E.	*P*	OR(95%CI)
Intercept	−3.260	0.649	<.0001	
Encapsulated effusion	0.970	0.697	0.164	2.639 (0.673–10.351)
Endometriosis/adenomyosis	1.729	0.651	0.008	5.637 (1.574–20.187)
Leiomyoma	−0.713	0.629	0.257	0.490 (0.143–1.683)

Notes: In multivariate analysis, the equation is of significance when the *p* value of the whole test less than 0.0001

## Discussion

Hysterectomy is one of the most commonly used surgical methods for gynecological benign diseases. Patients always hope that they will not receive reoperation for gynecological diseases when hysterectomy was performed, which is not the case. The occurrence of pelvic masses after hysterectomy is one of the most common causes for reoperation. 39% of women who underwent hysterectomy for benign gynecologic indications received elective bilateral salpingo-oophorectomy to prevent ovarian cancer [7]. Recent studies shown that prophylactic bilateral salpingectomy is helpful not only in preventing high-grade serous type ovarian cancer, but also in decreasing adnexal pathologies [8]. Question was raised subsequently on whether or not the fallopian tubes are resected during hysterectomy relate to the nature and origin of the subsequent pelvic masses. However, there is no consensus on whether bilateral fallopian tubes should be resected with hysterectomy for benign diseases in premenopausal women.

Total of 247 patients were enrolled in this study. In simple hysterectomy group, the most common chief complaints for the onset of pelvic mass were found by physical examination, abdominal distension and anorexia. Most common chief complaints in hysterectomy with salpingectomy group were found by physical examination and abdominal pain. 20.20% benign masses were derived from oviduct, and no malignant masses were derived from oviduct for the former group. Neither benign nor malignant masses were derived from oviduct in the latter group.

It has been thought that the fallopian tube has little physiological function after completing fertility, and may have potential of inducing cancer. More and more evidences show that ovarian cancer, especially high-grade serous carcinoma, originates from the fallopian tube. Malignant transformation of the fallopian tube can lead not only to fallopian tubal carcinoma, but also lead to the occurrence of ovarian and peritoneal carcinoma [2, 3]. Bilateral salpingectomy can reduce the risk of ovarian carcinoma for women with BRCA1/2 gene mutations or general population [9–12].

In addition, the retained fallopian tubes have the risk of secondary benign lesions, such as hydrosalpinx, fallopian tube ovarian abscess, fallopian tube prolapse, mesenchymal cyst of oviduct, etc. [13]. It has been reported that the risk of reoperation due to tubal diseases after hysterectomy without bilateral salpingectomy is twice as high as that with bilateral salpingectomy. Morse, et al. reported that women who underwent hysterectomy had 8% lifetime risk of reoperation for hydrosalpinx [14]. Another retrospective study reported that patients without preventive salpingectomy had a higher incidence of adnexal benign tumors (26.9% vs 13.9%, *P* = 0.02) [15]. The percentage of simple hysterectomy and hysterectomy with bilateral salpingectomy in our study is similar to those of Shiber Linda Dalal, et al. (80.16, and 5.67% respectively in the former one; 76, and 4% respectively in the latter one,) [16]. Shiber, et al. revealed that 82% of pelvic masses following hysterectomy are benign, and 18% were malignant. 12.4% were derived from fallopian tubes, accounting for 6.7% of benign masses and 12.9% of malignant masses. Therefore, the authors suggest that simultaneous salpingectomy with hysterectomy may reduce the incidence of benign tumors derived from the fallopian tubes. In this study, 68.2% of the pelvic masses in the simple hysterectomy group were benign and 31.8% were malignant. Tubular masses accounted for 16.2% of the total pelvic masses and 21.5% of the benign masses, and there were no malignant tumors. In the simultaneous salpingectomy group, 57.1% of the pelvic masses were benign and 42.9% were malignant with none of the masses derived from fallopian tube. Therefore, the results of this study are in accordance to the results of Shiber that hysterectomy combined with simultaneous bilateral salpingectomy may reduce the incidence of fallopian tubes derived pelvic masses, which accounted for 12.4–16.2% of total pelvic masses and 6.7–21.5% of total benign pelvic masses.

Furthermore, we compared the clinicopathological data of patients who underwent hysterectomy with and without simultaneous bilateral salpingectomy in this study. After adjusting the factors of more patients with endometriosis or adenomyosis in salpingectomy group, we found that there was no statistical difference between two groups on the age of pelvic mass onset, time interval between twice operations, main complaints when pelvic mass was found, and proportion of benign and malignant tumors. We also found that salpingectomy lead to no difference on the malignant pelvic masses between two groups, but cause difference on the benign pelvic masses. On the one hand, there were fewer cases with salpingectomy in this study, which may affect the statistical results. On the other hand, the objects of this study were the patients who underwent reoperation due to pelvic mass after simple hysterectomy with and without simultaneous bilateral salpingectomy, and we did not enrolled all patients underwent hysterectomy. Therefore, result of this study cannot concluded that simultaneous salpingectomy cannot reduce the incidence of benign and malignant masses, and cannot make difference on the nature of the pelvic masses. The prospective study we are currently conducting may help explaining this issue.

## Conclusion

In conclusion, the results of this study showed that 14.65% of the pelvic masses in the hysterectomy group originated from fallopian tubes, accounting for 21.48% of the benign masses, while the pelvic masses in the salpingectomy group had no tumors originating from fallopian tubes. Therefore, hysterectomy with simultaneous bilateral salpingectomy may reduce the recurrence of pelvic masses, especially benign pelvic masses. However, there were no significant differences in clinical features and pathological types between the two groups.

## Abbreviations

IQR: Interquartile range
ROC: Receiver operating characteristic curve
